# Long-term complete remission with immunotherapy in advanced RET fusion-positive NSCLC with brain metastases: a case report and literature review

**DOI:** 10.3389/fonc.2025.1597110

**Published:** 2025-09-11

**Authors:** Shuheng Shang, Meng Qin, Shuangmei Zhang

**Affiliations:** ^1^ Department of Radiotherapy, Huzhou Central Hospital, Fifth Affiliated Clinical Medical College of Zhejiang Chinese Medical University, Hangzhou, China; ^2^ Department of Drug Clinical Trial Institution, Huzhou Central Hospital, Fifth Affiliated Clinical Medical College of Zhejiang Chinese Medical University, Hangzhou, China; ^3^ Department of Respiratory, Huzhou Central Hospital, Fifth Affiliated Clinical Medical College of Zhejiang Chinese Medical University, Hangzhou, China

**Keywords:** RET fusion-positivity, NSCLC, immunotherapy, radiotherapy, brain metastases

## Abstract

The rearranged during transfection (RET) fusion is a rare genetic alteration in non-small-cell lung cancer (NSCLC), and the presence of brain metastases significantly influences prognosis. We present a 59-year-old patient diagnosed with RET fusion-positive lung adenocarcinoma which had metastasized to the brain at the time of initial diagnosis, classified as stage IVB, cT3N3M1. Tumor biopsy immunohistochemistry showed PD-L1 positivity (10%). After three cycles of pemetrexed plus cisplatin (PC) regimen combined with camrelizumab (a PD-1 inhibitor), a partial response (PR) was observed through chest computed tomography (CT) and brain magnetic resonance imaging (MRI). The patient underwent whole brain radiotherapy (WBRT) with a total dose of 37.5 Gy over 15 fractions, followed by 3 cycles of the PC regimen plus camrelizumab. Complete remission (CR) was achieved during 30 months of maintenance therapy with pemetrexed plus camrelizumab. The most recent follow-up was in February 2025. Both chest CT and brain MRI continued to show CR, with no clear indications of metastases. During the course of immunochemotherapy, grade 1 bone marrow suppression, but no toxicity of grade 3 or above, was observed. NSCLC patients who have PD-L1 overexpression and RET fusion-positivity may respond well to immunotherapy. Combining radiotherapy with immunotherapy may enhance local control of brain metastases.

## 1 Introduction

The RET proto-oncogene is located on chromosome 10 and encodes a transmembrane receptor tyrosine kinase that is involved in the development of the renal, nervous, and respiratory systems (1). Mutations in the RET gene or fusions with other chromosomal domains can directly or indirectly activate the kinase, leading to spontaneous ligand-independent signaling, uncontrolled cell growth, and malignant tumor development (2). Next-generation sequencing identified KIF-5B (78.9%) and CCDC6 (15.8%) as the most frequent fusion partner genes of RET (3, 4). Somatic mutations in the RET gene are common in both sporadic and radiation-induced papillary thyroid carcinomas (5). In 2012, RET fusions were first identified in lung cancer, with an incidence of merely 1%-2% (6). Similar to ALK and ROS1 rearrangements, RET fusions are prevalent in younger and never-smoking NSCLC patients. Most of these adenocarcinomas are poorly differentiated and advanced at the time of diagnosis, leading to a poor prognosis (7, 8). This study presents an unusual case of RET fusion-positive NSCLC with brain metastases that achieved full remission of both the primary tumor and the brain metastases.

## 2 Case report

**Figure 1:**
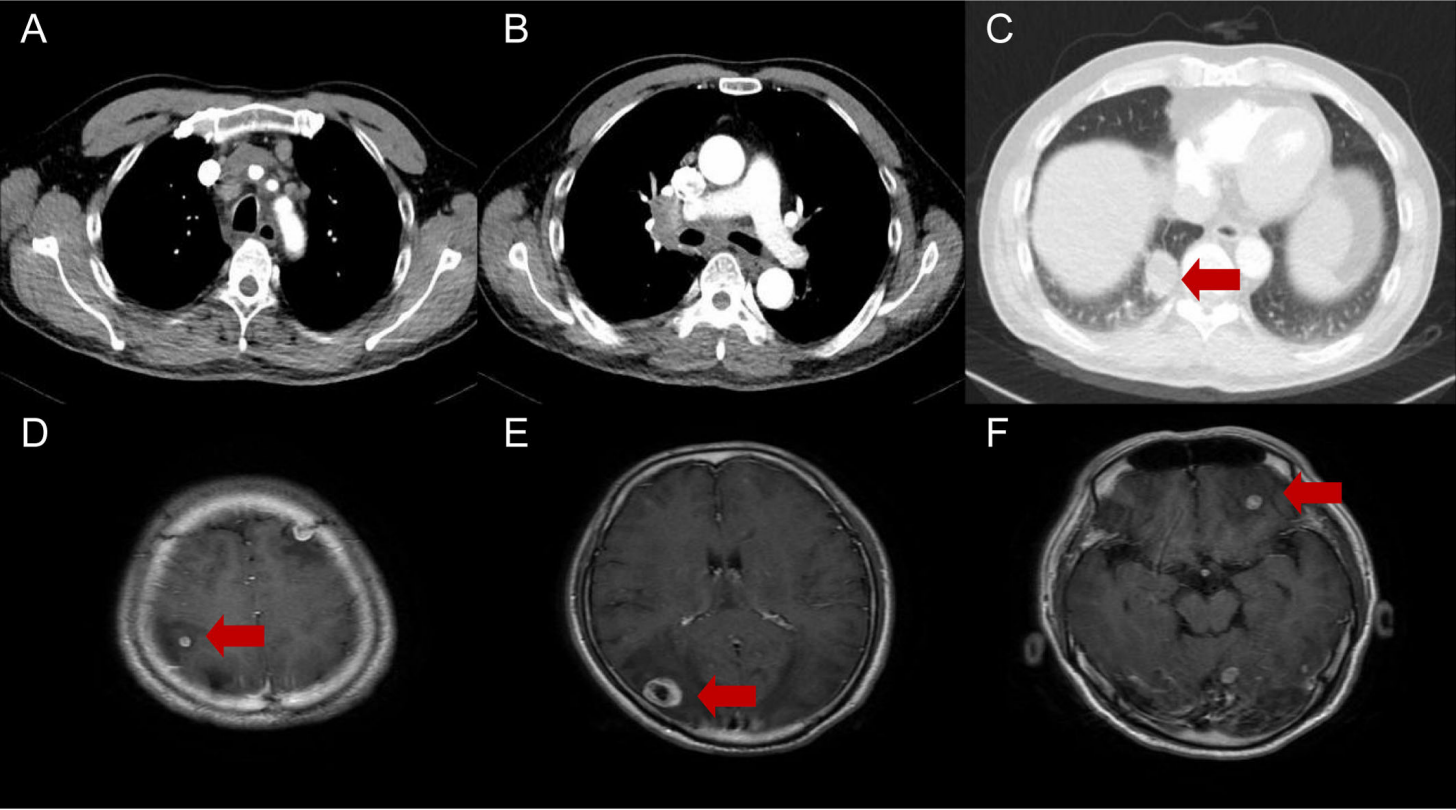
Diagnostic chest CT and brain MRI of a 59-year-old patient indicating primary lung cancer **(A-C)** and brain metastases **(D-F)**. The arrows indicate a 33×30×28 mm mass in the inferior lobe of the right lung and multiple intracranial metastases.

**Figure 2:**
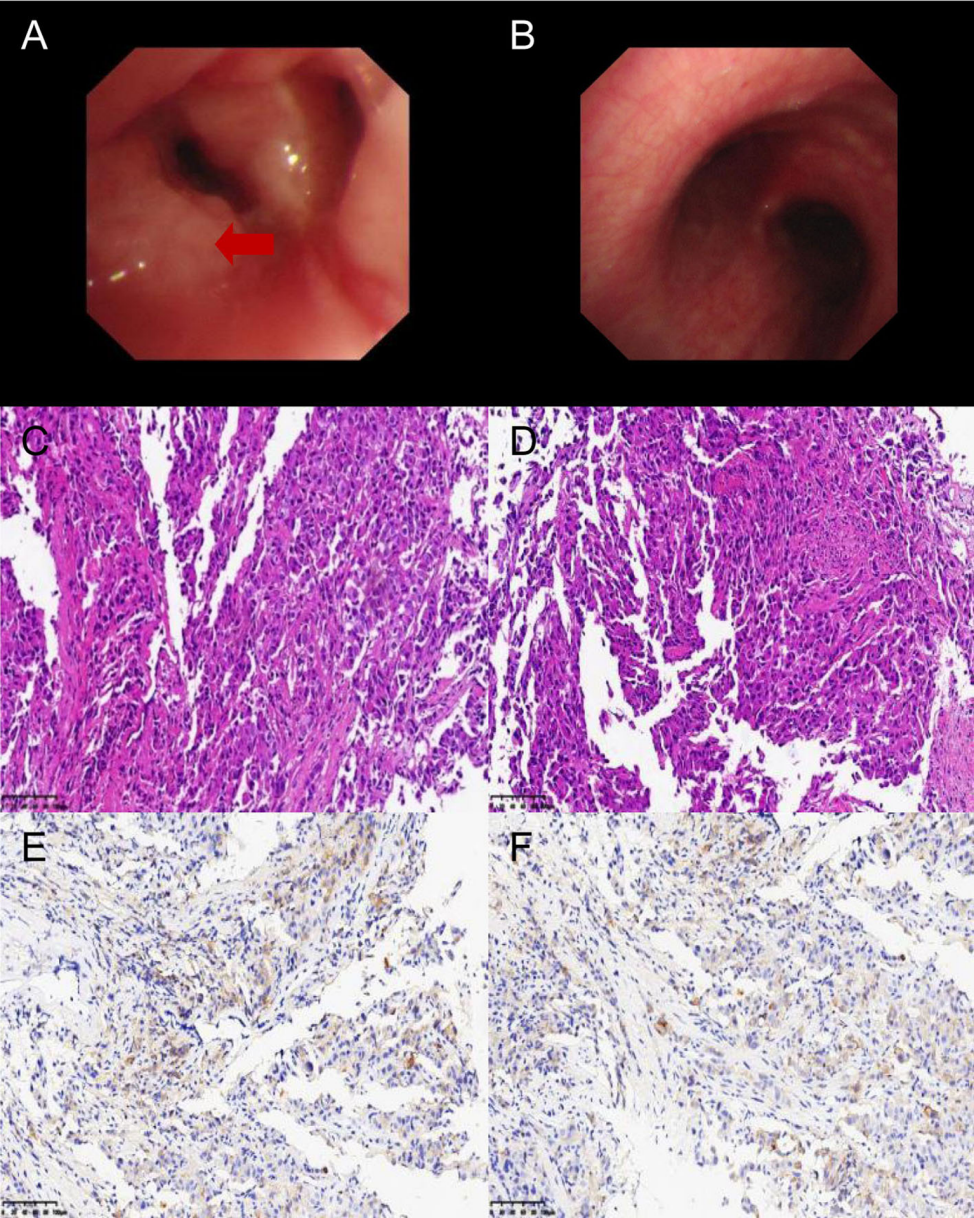
Bronchoscopy **(A, B)**, HE **(C, D)**, and immunohistochemical (PD-L1) staining of the primary lung lesion **(E, F)**. Bronchoscopy identified a mass (the red arrow) at the opening of the basal segment of the right lower lobe. PD-L1 expression = 10% (200×).

**Figure 3:**
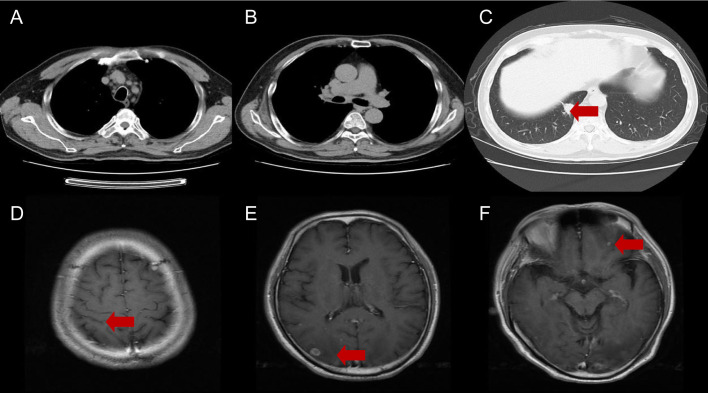
Chest CT **(A-C)** and brain MRI **(D-F)** after 3 cycles of immunochemotherapy. The red arrows indicate the responses in both lung cancer and brain metastases.

**Figure 4:**
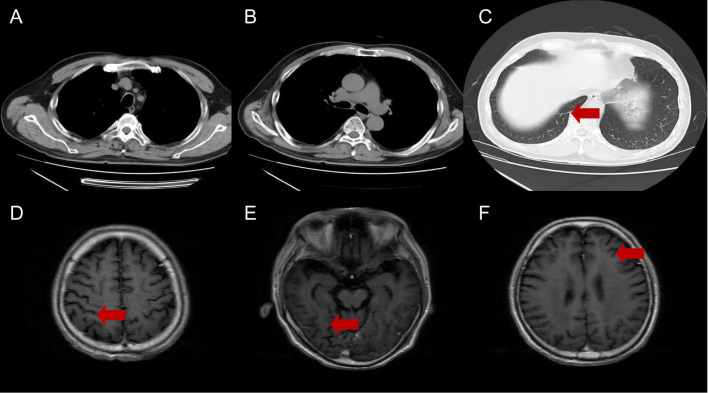
Chest CT **(A-C)** and brain MRI **(D-F)** at last follow-up. The red arrows indicate complete regressions in both lung cancer and brain metastases.

A 59-year-old man visited our hospital on September 8, 2021, manifesting dizziness and blurred vision over the last month. Multiple low-density areas with nodules were observed in the brain parenchyma through an enhanced MRI. A contrast-enhanced CT scan of the chest showed a mass, suspected to be a malignant tumor, and possible obstructive inflammation in the right lower lung lobe. Multiple enlarged lymph nodes were also noted in the right hilum and mediastinum (**Figure 1**). Contrast-enhanced abdominal CT and other supplementary tests indicated no further evidence of metastases. Hormones were used to alleviate cerebral edema, and treatments for dizziness and high blood pressure were given. During bronchoscopy, a mass was discovered at the basal segment’s opening in the right lung’s lower lobe. The biopsy identified it as poorly differentiated carcinoma. The immunohistochemistry profile revealed CK5/6+, P40-, CK7+, TTF1+, NapsinA+, Ki-67+ (60%), and PD-L1+ (TPS 10%), confirming a histological diagnosis of adenocarcinoma (**Figure 2**). Multigene testing showed that the patient was positive for RET-Exon-12 fusion. Due to financial reasons, the patient declined the RET inhibitor platinib. A PC regimen along with camrelizumab (an anti PD-1 antibody) was provided for three cycles after ensuring no contraindications. On October 20, 2021, chest CT and brain MRI demonstrated a substantial decrease in the size of the primary lung tumor and a reduction in the number of brain metastatic nodules (**Figure 3**). WBRT began on October 25, 2021. Intensity modulated radiotherapy techniques were adopted. Clinical target volume encompassed the entire brain parenchyma. Planning target volume was defined by applying a 3 mm margin to the CTV. The prescribed radiation dose was 37.5 Gy, delivered in 15 fractions, in compliance with dose/volume constraints for organs at risk. Following an additional three cycles of the PC regimen plus camrelizumab, maintenance therapy with pemetrexed and camrelizumab was initiated, and its effectiveness was evaluated through regular examinations. CR of the primary lung lesion was observed in chest CT scans on October 21, 2022, and brain MRI imaging demonstrated CR of brain metastases on April 6, 2023. The last maintenance treatment was performed on August 16, 2024. No signs of further metastases were observed during the last follow-up in February 2025 (**Figure 4**). Hypothyroidism emerged a year post-immunotherapy and was addressed with oral levothyroxine. Throughout the treatment, the patient experienced grade 1 bone marrow suppression, with no instances of grade 3 or higher toxicity.

## 3 Discussion

About 46% of NSCLC patients with RET fusion develop brain metastases (9), for which systemic therapy combined with local radiotherapy is the recommended treatment approach. The patient reported herein had symptomatic multiple brain metastases at the time of diagnosis. Currently, treatments targeting RET fusion comprise mainly TKIs. However, multi-target TKIs (mTKIs) such as capotinib, vandetanib, and lenvatinib not only lack specificity, but also heighten off-target effects. In an international multicenter study including 165 patients with RET fusion-positive NSCLC, 53 were treated with one or more mTKIs, resulting in a median progression-free survival (PFS) of merely 2.3 months and a median OS of 6.8 months (10). Selpercatinib and pralsetinib, two highly selective small molecule RET kinase inhibitors (RETi), have shown promising antitumor activity and safety (11, 12). Moreover, as these targeted drugs can penetrate the blood-brain barrier (BBB), they have a significantly better ability to reduce tumor burden in the central nervous system than traditional chemotherapy (13). The LIBRETTO-001 trial, a Phase 1/2, single-arm, open-label study of selpercatinib for RET fusion-positive NSCLC patients, reported an 84% objective response rate (ORR), with 6% of patients achieving CR, a median response duration of 20.2 months, and a median PFS of 22.0 months. Among the 26 patients with measurable brain metastases, the intracranial ORR was 85%, with a CR rate of 27% (14). In the Phase 1/2 ARROW trial, 233 patients with RET fusion-positive NSCLC were treated with pralsetinib. ORR was 61%. Grade 3/4 drug-related toxicities included neutropenia (18%), hypertension (11%), and anemia (10%), with no treatment-related deaths (15). The RET-MAP multicenter retrospective study enrolled 218 patients with RET fusion-positive NSCLC who received, alternatively, monotherapy, doublet chemotherapy, immune checkpoint inhibitors (ICIs), chemotherapy combined with ICIs, mTKIs, and RETi. Brain metastases were detected in 31% of cases, and the median PFS after the above treatments was 3.6, 8.7, 3.1, 9.6, 3.1, and 16.2 months, respectively. Of note, patients treated with RETi had a longer median OS compared to those who did not (50.6 months versus 16.3 months, P < 0.0001) (16). For economic reasons, the patient refused RETi treatment. However, after 3 cycles of combination chemoimmunotherapy, the brain metastases and primary lesions were significantly diminished. Following WBRT, maintenance immunotherapy was carried out for 30 months. CR was successively achieved for both intracranial metastases and primary lesions with good tolerance.

Prior to the development of targeted drugs, platinum-based double-agent chemotherapy was the standard treatment for advanced RET fusion-positive NSCLC. Shen et al. analyzed 62 cases of stage IIB/IV RET fusion-positive NSCLC, including 41 KIF5B-RET, 15 CCDC6-RET, and 6 other rare fusion-positive subtypes. The median PFS and OS for patients on pemetrexed-based chemotherapy were 9.2 months and 35.2 months, respectively, contrasting with 5.2 months and 22.6 months for those on other chemotherapy treatments (17). European studies revealed an ORR of 45% and a median PFS of 19 months for pemetrexed-based chemotherapy, highlighting its enduring efficacy, similar to that observed in ALK and ROS1 fusion-positive NSCLC (18). Regional differences in pemetrexed’s effectiveness may be attributed to population heterogeneity, with a greater number of smokers among European compared to Asian patients (19).

Because the BBB prevents macromolecular chemotherapy drugs from attaining sufficient levels in the brain, local radiotherapy is generally the standard treatment for brain metastases. For multiple brain metastases, WBRT is the main choice and can also serve as a consolidation treatment following surgery or stereotactic radiosurgery (SRS). Dose fractionation schedules usually involve 30-40 Gy in 10-20 fractions. While WBRT can enhance intracranial local control rates, a study involving NSCLC patients with brain metastases unsuitable for surgical resection or stereotactic radiotherapy concluded that WBRT does not improve OS compared to optimal supportive care, pointing also that its long-term neurotoxic effects can lead to significant cognitive impairments and reduced quality of life (20). The case reported here achieved a CR, with a PFS of more than 42 months. Considering the limitations of pemetrexed chemotherapy and WBRT in brain metastases treatment, we suspect that the improvement in PFS is probably associated with anti-PD-1 immunotherapy.

The standard protocol for advanced NSCLC is based on ICIs therapy; however, NSCLC with driver gene mutations is characterized by low immunogenicity. In such cases, the presence of immunosuppressive cells and the release of immunosuppressive substances together create a ‘forbidden zone’ for ICIs (21). A retrospective analysis of 551 lung cancer patients from 24 centers in 10 countries revealed that in the 16 patients with RET fusion-positive NSCLC, ICI monotherapy achieved an ORR of only 6% and a median PFS of 2.1 months, which were overall lower compared to the responses of patients with other driver mutations such as EGFR, KRAS, and BRAF (22). Offin et al. reported 74 RET fusion-positive NSCLC cases treated with single or dual-agent ICIs. No immune response was noted in the 13 patients evaluated for efficacy, 62% (8/13) of them were identified with progressive disease, and no connection was seen between PFS and PD-L1 expression or TMB status. Even in the 2 patients with high PD-L1 expression (50% and 30%, respectively), no PFS benefit was seen (1.3 months and 2.5 months, respectively) (23).

Nevertheless, recent studies indicated that patients with RET fusion and high PD-L1 expression may benefit from ICIs. In the phase 3 LIBRETTO-431 trial, researchers assessed serpatinib versus platinum-based chemotherapy (with or without pembrolizumab) for first-line treatment of RET fusion-positive NSCLC. Interim results indicated a median PFS of 24.8 months for serpatinib, compared to 11.2 months for the control group, with objective response rates of 84% and 65%, respectively. Importantly, the outcomes observed in the control group were similar or better than those reported in the KEYNOTE-189 trial (24). Lu et al. enrolled 129 patients with RET fusion-positive NSCLC who received chemotherapy, ICIs, or mTKIs. Although the median PFS was not significantly different among the three groups (3.5 months, 2.5 months, and 3.8 months, respectively), in the ICI-treated patients the disease control rate was 60% (6/10), the ORR was 20% (2/10), and two patients achieving PR showed high PD-L1 expression (TPS ≥ 50%) (25). A study by Guisier et al. included nine RET fusion-positive NSCLC patients with prior systemic therapy who received single-agent ICIs. Two patients had PD-L1 expression ≥ 50%, five were PD-L1-negative, and the ORR was 37.5% (26). Bhandari et al. found that among 69 patients with RET fusion-positive NSCLC treated with immunotherapy with or without chemotherapy, the median PFS was 4.2 months and the median OS was 19.1 months. This survival benefit was indeed consistent with literature reports for unspecified populations (27). However, since the studies mentioned above are limited by their small sample size, whether RET fusion-positive NSCLC patients would benefit from ICI treatment is still controversial.

Although immune checkpoint inhibitors have shown effectiveness in some RET fusion-positive subtypes, the precise mechanism remains poorly understood. RET fusion-positive NSCLC mostly present as an “immune cold” phenotype with low tumor mutational burden, low PD-L1 expression, and limited immune cells (22). In such low-immunogenic tumors, combination regimens such as chemotherapy, targeted therapy, radiotherapy, or other immunomodulatory therapies may improve the immunosuppressive tumor microenvironment. These strategies can also decrease regulatory T cells and tumor-associated macrophages or alleviate hypoxia, potentially boosting immunotherapy effectiveness (28, 29).Identifying those who may respond to ICIs remains a current challenge. Although PD-L1 serves as a biomarker for immunotherapy in NSCLC, its predictive potency is constrained. The correlations between treatment response rates and longer progression-free survival or overall survival were found to be weak to moderate when evaluated by PD-L1 expression levels (30, 31). In the future, the integration of blood biomarkers, radiomics, and gut microbiome may hold promise for optimizing treatment strategies.

The brain is considered an immune-privileged organ, with the BBB protecting brain tissue from attack by immune cells (32).However, brain metastases disrupt the BBB and facilitate the invasion of immune cells from the peripheral circulation (33). Single-cell RNA sequencing revealed that lung cancer brain metastases are infiltrated with a variety of immune cells including microglia, macrophages, mast cells, and CD8+ T cells, albeit these cells were functionally suppressed (34). Due to concerns about low intracranial penetration, potential toxicity, and shortened life expectancy, clinical trials of ICIs often excluded patients with brain metastases (35). However, recent studies suggested that ICIs may be indeed effective to treat brain metastases, whether administered alone or in combination with chemotherapy or radiotherapy. In a meta-analysis of KEYNOTE-021, 189, and 407 trial results, among 171 patients with NSCLC and stable brain metastases at baseline those who received pembrolizumab plus chemotherapy showed improved PFS and OS (6.9 months vs 4.1 months and 18.8 months vs 7.6 months, respectively) compared to patients treated with chemotherapy alone (36). At present, there are two main hypotheses regarding the validity of ICIs treatment for brain tumors: one states that systemic immune activation by ICIs leads to parallel activation of lymphocytes in the brain; the other poses that damage to the BBB allows ICIs to penetrate the central nervous system (37). Although the exact mechanism remains unclear, promising clinical outcomes led to the emergence of ICIs as a first-line treatment option for brain metastases in advanced NSCLC.

The role of combination of immunotherapy and radiotherapy in the treatment of brain metastases is also a topic of great interest. Preliminary studies suggest that radiotherapy can boost immunotherapy effectiveness through a variety of mechanisms, such as promoting the release of tumor-associated antigens by immunogenic cells, increasing the number of cytotoxic T lymphocytes, increasing the permeability of the BBB, increasing PD-L1 expression in tumor cells, and promoting M2 to M1 transition in macrophages (38, 39). A phase 2 single-arm prospective study found that NSCLC patients with brain metastases treated with altelizumab, carboplatin, and pemetrexed had a median PFS of 8.9 months and an intracranial PFS of 6.9 months, demonstrating satisfactory safety and efficacy. Radiotherapy was administered to most patients after intracranial progression, implying the practicality of combining immunotherapy with delayed radiotherapy (40). The effectiveness and safety of combining SRS and ICIs are also under investigation. In a small clinical study involving 13 NSCLC patients with active brain metastases, the median intracranial PFS for SRS in conjunction with nivolumab/ipilimumab was 9.7 months and the intracranial PFS rate at 4 months was 70.7%. However, 3/13 patients experienced grade 3 or higher treatment-related adverse events, including elevated transaminases, fatigue, nausea, adrenal cortical insufficiency, and myocarditis (41). These findings reinforce the need to evaluate optimal timing and dose fractionation for brain metastasis-targeted radiotherapy, especially when applied in combination with immunotherapies.

## 4 Conclusion

To our knowledge, this is the first case report to achieve CR and an unusually long PFS after receiving immunotherapy for advanced RET fusion-positive NSCLC. Immunotherapy serves as an optional approach for NSCLC with positive driver genes, especially when PD-L1 expression is high, and increased benefits may be obtained upon its combination with chemotherapy or radiotherapy.

## Data Availability

The original contributions presented in the study are included in the article/supplementary material. Further inquiries can be directed to the corresponding author.
